# Constructing core competency indicators for clinical teachers in Taiwan: a qualitative analysis and an analytic hierarchy process

**DOI:** 10.1186/1472-6920-14-75

**Published:** 2014-04-11

**Authors:** Ai-Tzu Li, Jou-Wei Lin

**Affiliations:** 1 Institute of Adult and Continuing Education, National Chung-Cheng University, 168 University Road, Min-Hsiung (621), Chia-Yi County, Taiwan; 2 Department of Medicine, College of Medicine, National Taiwan University, Taipei (100), Taiwan; 3 Department of Internal Medicine, National Taiwan University Hospital Yun-Lin Branch, 579 Yun-Lin Road, Section 2, Dou-Liou City (640), Yun-Lin County, Taiwan

**Keywords:** Medical education, Clinical teachers, Core competency, Questionnaires, Analytic hierarchy process

## Abstract

**Background:**

The objective of this study was to construct a framework of core competency indicators of medical doctors who teach in the clinical setting in Taiwan and to evaluate the relative importance of the indicators among these clinical teachers.

**Methods:**

The preliminary framework of the indicators was developed from an in-depth interview conducted with 12 clinical teachers who had previously been recognized and awarded for their teaching excellence in university hospitals. The framework was categorized into 4 dimensions: 1) Expertise (i.e., professional knowledge and skill); 2) Teaching Ability; 3) Attitudes and Traits; and 4) Beliefs and Values. These areas were further divided into 11 sub-dimensions and 40 indicators. Subsequently, a questionnaire built upon this qualitative analysis was distributed to another group of 17 clinical teachers. Saaty’s eigenvector approach, or the so-called analytic hierarchy process (AHP), was applied to perform the pairwise comparisons between indicators and to determine the ranking and relative importance of the indicators.

**Results:**

Fourteen questionnaires were deemed valid for AHP assessment due to completeness of data input. The relative contribution of the four main dimensions was 31% for Attitudes and Traits, 30% for Beliefs and Values, 22% for Expertise, and 17% for Teaching Ability. Specifically, 9 out of the 10 top-ranked indicators belonged to the “Attitudes and Traits” or “Beliefs and Values” dimensions, indicating that inner characteristics (i.e., attitudes, traits, beliefs, and values) were perceived as more important than surface ones (i.e., professional knowledge, skills, and teaching competency).

**Conclusion:**

We performed a qualitative analysis and developed a questionnaire based upon an interview with experienced clinical teachers in Taiwan, and used this tool to construct the key features for the role model. The application has also demonstrated the relative importance in the dimensions of the core competencies for clinical teachers in Taiwan.

## Background

Medical education plays a critical role in the professional development of a physician; however, the expertise of the clinical teachers who instruct and direct medical education is rarely discussed. In today’s fast-paced, technologically-advanced society, medical professionals are facing significant challenges, such as changing features of medical practice, decreased returns and increased burden, complicated working environments, and conflicting interests among doctors, patients, and payers [1]. Medical students and residents often deal with issues surrounding morals and ethics [2]. Current medical training does not provide enough education in these areas. Medical education must adapt to these changing times, and clinical teachers must lead the charge.

Despite an integrated educational reform might be needed to cultivate the new generation of physicians, only few teaching methods exist in the medical education systems in Taiwan. The frequently applied teaching tools in this southeast Asian country are usually limited to: 1) basic medical classes taught in a big classroom setting; 2) small group discussions on current medical issues involving 7 or 8 medical students and a clinical teacher; and 3) clinical practice in each specialty to gain professional knowledge, skills, and experiences, and to develop and establish attitudes and values [3]. All three types of medical education contribute significantly to the development of a medical professional in Taiwan. Medical students learn to apply the theories discussed in the classroom to patients and develop attitudes toward the patients.

Hsieh, one of Taiwan’s leading medical educators, stated that good clinical teachers should support an educational philosophy with enthusiasm in and devotion to teaching, and sound teaching content and methods. They play an extremely important role in cultivating appropriate professional behavior in the medical students [4]. However, it is reported that clinical teachers in Taiwan lacked teaching skills because medical schools did not have enough resources or support to facilitate improvement in their teaching. Furthermore, little was known about how to adequately evaluate their teaching performance [5]. In the meantime, only 55.9% of students were confident that they had acquired the clinical skills required to become a resident, and 70.7% were satisfied with the quality of their medical education [6].

One of the major reasons behind this situation is that we have believed in false for a long time that medical doctors will become good teachers once they have sufficient clinical experience [7]. The core competencies required for the medical doctors who must also play a role as clinical teachers are seldom discussed. Therefore, it is urgent and important to develop a framework that incorporates both the teaching dimensions and clinical abilities that formulate the core competencies of clinical teachers in Taiwan. The objective of this study was thus to explore the components of core competency of clinical teachers in Taiwan, to develop an indicator framework of our own, and to examine the relative importance of these indicators.

## Methods

We conducted a literature review on the definition and meaning of “core competency” with a focus on the job description, roles, and inner and surface characteristics of clinical teachers [8-20]. The literature review provided the lenses for analyzing the data. The methods included two stages: in-depth interview and the analytic hierarchy process (AHP). The interviews were approved by the Research Ethics Committee of National Taiwan University Hospital (IRB# 201101050RC).

First, we conducted an in-depth interview with experienced clinical teachers in Taiwan. Those medical doctors were selected from different teaching hospitals from the northern, middle, and southern parts of Taiwan. The inclusion criteria involved 1) having clinical teaching experiences more than 3 years, 2) receiving teaching excellence awards from university hospitals or the government, and 3) being willing to attend the study. We first sent out invitation letters to the clinical teachers who fulfilled the criteria and obtained 12 clinical teachers. We then performed a qualitative analysis based upon the audio-taped records. Data were analyzed from the first interviewee and on an ongoing analysis. When the data was saturated, we stopped inviting interviewees [21]. Constant comparative method was utilized to analyze the data [22]. We first read the transcripts thoroughly in order to get a whole picture of the data. Then, we tried to categorize the concepts and build the relationship among categories. Further, we identified the meanings and themes from the data. We constantly compared earlier data with later ones in the data analysis process. A framework composed of four main dimensions: 1) Expertise, 2) Teaching Ability, 3) Attitudes and Traits, and 4) Beliefs and Values, sub-dimensions, and underlying indicators was established. Further, a questionnaire based upon this framework was constructed (Table 1).

**Table 1:** The indicator framework of core competencies for clinical teachers

**Dimensions**	**Sub-dimensions**	**Behavioral indicators**
Expertise	Clinical abilities	Being equipped with clinical knowledge
		Ability to make the correct judgment
		Possessing clinical handling ability
		Being familiar with clinical ethics, regulations, and systems
		Possessing concept of whole medicine
	Clinical communication	Ability to explain the illness to the patient
		Ability to use language the patient and his/her family understands
		Ability to handle difficult situations
		Ability to clearly explain the treatment plan, including risks and benefits
		Ability to listen to the patient’s and family’s needs and demands
		Ability to be sympathetic to the patient and the family
		Ability to communicate and cooperate with the medical team
Teaching Ability	Instructional design	Ability to set up learning goals
		Ability to use proper teaching methods according to learning goals
		Ability to arrange proper teaching content according to students’ characteristics
	Teaching skills	Ability to guide students’ thoughts and discussion
		Ability to establish a lively and active teaching atmosphere
		Ability to provide examples that are close to students’ experience
		Ability to give students chances to practice
		Ability to introduce clinical cases by empirical medicine method
	Teaching material design	Designing or selecting proper teaching materials by learning goals
		Ability to transform clinical cases to teaching materials
		Ability to use multimedia teaching materials
	Learning evaluation	Ability to collect related evaluation data
		Ability to use proper evaluation tools correctly
		Ability to review one’s teaching after evaluation
Attitudes and Traits	Teaching attitudes	Enthusiastic teaching
		Ability to interact well with students
		Ability to support and help students anytime
		Ability to guide students in their lives
	Professional growth	Ability to absorb new medical information anytime
		Ability to take part in various kinds of teaching workshops
		Ability to publish research paper regularly
	Personality traits	Being friendly and kind
		Patience
		Being confident in self-expression
Beliefs and Values	Teaching beliefs	Ability to identify the implications and values of teaching
		Ability to pass on medical knowledge
	Model teaching	Ability to teach by words and set an example
		Acting as a role model for young teachers

In the next step, clinical teachers in five teaching hospitals in the northern, middle, and southern parts of Taiwan were invited to evaluate the professionals’ questionnaires. In order to thoroughly understand the relative importance of each indicator, there were three sources of research subjects: 1) senior clinical teachers with academic reputations in the medical education domain; 2) clinical teachers who serve as the teaching-type attending doctors; and 3) teachers recommended by the Director of Teaching and Learning Development Center. Questionnaires were sent to these clinical teachers once they accepted the invitation. The questionnaires were deemed effective when all data input were complete to make pairwise comparison.

To understand the relative importance of each indicator, we adopted the analytic hierarchy process (AHP) developed by Saaty to build up a physical analysis of the relative weight of each core competency indicator for clinical teachers [23,24]. According to Triantaphyllou and Mann, “the AHP is a decision support tool which uses a multi-level hierarchical structure of objectives, criteria, subcriteria, and alternatives. The pertinent data are derived by using a set of pairwise comparisons. These comparisons are used to obtain the weights of importance of the decision criteria, and the relative performance measures of the alternatives in terms of each individual decision criterion”[25].

The questionnaires were presented by paired indicators. For each question, both sides had a factor, and the more important one was selected first by the professionals. Then, the score scale of the relative importance (1–9) was determined. A sample question (e.g., What do you think about the relative importance of the two behavioral indicators of expertise [clinical ability and professional communication]? Please check the proper field and then determine the relative importance.) is shown in Table 2. After collecting the questionnaires, the AHP module matrix written with Excel was utilized to conduct data analysis, and each question was checked to see whether or not it met a consistency check (shown by consistency ratio, or the CR values). If the CR value was <0.1, the research result was effective [25].

**Table 2:** A sample question in the questionnaire

**Behavioral indicator**	**1**	**2**	**3**	**4**	**5**	**6**	**7**	**8**	**9**	**Behavioral indicator**
□ Clinical ability										□ Professional communication

Note:

1 Equal importance.

3 Moderate importance of one over another.

5 Essential or strong importance.

7 Demonstrated importance.

9 Absolute importance.

2, 4, 6, 8 Intermediate values between the two neighboring scales.

## Results

The framework developed by the first group of 12 clinical teachers included four dimensions (Expertise, Teaching Ability, Attitudes and Traits, and Beliefs and Values), 11 sub-dimensions, and 40 indicators (Table 1).

Another group of 25 clinical teachers in the teaching hospitals served as the AHP questionnaire evaluating professionals. After sending the invitation letters via e-mail, 17 professionals agreed to participate in this research. Sixteen of 17 questionnaires were returned (response rate 94%), and 14 questionnaires were effective due to data completeness (return rate 87%). Ten questionnaires came from men (71%), and four questionnaires came from women (29%). As for the teaching experience, one teacher taught <5 years (7.1%); 4 teachers taught between 5 and 10 years (28.6%); 3 teachers taught between 10 and 20 years (21.4%); and 6 teachers taught >20 years (42.9%).

Matrix and weight analysis of the four dimensions of the second hierarchy (Expertise, Teaching Ability, Attitudes and Traits, and Beliefs and Values) are shown in Table 3. Among the four dimensions of clinical teachers’ core competency, “Attitudes and Traits” was perceived as more important than “Beliefs and Values,” “Expertise,” and “Teaching Ability.” The relative contribution was 31%, 30%, 22%, and 17%, respectively.

**Table 3:** Analysis of matrix and weight of the four dimensions

**Dimensions**		**F1**	**F2**	**F3**	**F4**	**Weight**	**Rank**
Expertise	F1	1	2.037	0.645	0.501	22%	3
Teaching Ability	F2	0.490	1	0.516	0.793	17%	4
Attitudes and Traits	F3	1.547	1.937	1	1.067	31%	1
Beliefs and Values	F4	1.994	1.259	0.936	1	30%	2

CR = 0.043 < 0.1.

The sub-dimension analysis showed that in the Expertise dimension, “Clinical Communication” was perceived as more important than “Clinical Abilities” (within-dimensional weight: 53% and 47%, respectively) (Table 4A). Among the sub-dimensions of Teaching Ability, the relative importance was “Teaching Skills” (39%), followed by “Instructional Design” (27%), “Learning Evaluation” (22%), and “Teaching Material Design” (12%) (Table 4B). Among the sub-dimensions of Attitudes and Traits, the relative importance was “Teaching Attitudes” (50%), followed by “Personality Traits” (26%), and then “Professional Growth” (24%) (Table 4C). In the dimension of Beliefs and Values, “Model Teaching” (61%) was perceived as more important than “Teaching Beliefs” (39%) (Table 4D).

**Table 4:** Analysis of matrix and weight of expertise dimension (panel A), teaching ability dimension (panel B), attitudes and traits dimension (panel C), and beliefs and values dimension (panel D)

**Dimensions**	**Sub-dimensions**							
A. Expertise			F1	F2			Weight	Rank
	Clinical Abilities	F1	1	0.885			47%	2
	Clinical Communication	F2	1.128	1			53%	1
CR = 0 < 0.1								
B. Teaching Ability			F1	F2	F3	F4	Weight	Rank
	Instructional Design	F1	1	0.865	1.833	1.209	27%	2
	Teaching Skills	F2	1.154	1	3.592	2.029	39%	1
	Teaching Material Design	F3	0.545	0.278	1	0.523	12%	4
	Learning Evaluation	F4	0.826	0.492	1.908	1	22%	3
CR = 0.011 < 0.1								
C. Attitudes and Traits			F1	F2	F3		Weight	Rank
	Teaching Attitudes	F1	1	2.116	1.986		50%	1
	Professional Growth	F2	0.472	1	0.917		24%	3
	Personality Traits	F3	0.503	1.089	1		26%	2
CR = 0 < 0.1								
D. Beliefs and Values			F1	F2			Weight	Rank
	Teaching Beliefs	F1	1	0.639			39%	2
	Model Teaching	F2	1.564	1			61%	1
CR = 0 < 0.1								

The relative weights of all behavioral indicators beneath the 11 sub-dimensions among the four dimensions are shown in Table 5. The 10 behavioral indicators with the highest ranks were 1) teaching by words and setting an example; 2) passing on medical knowledge; 3) acting as a role model for young teachers; 4) identifying the implications and values of teaching; 5) enthusiastic teaching; 6) ability to interact well with students; 7) ability to absorb new medical information anytime; 8) patience; 9) ability to support and help students anytime; and 10) possessing clinical handling ability. The top four indicators fell into the “Beliefs and Values” dimension; numbers 5–9 into the “Attitudes and Traits” dimension; and only number 10 into the “Expertise” dimension.

**Table 5:** Weight analysis of overall indicators

**Dimensions**		**Sub-dimensions**		**Behavioral indicators**				
	**Weight**		**Weight**		**Weight**	**Rank**	**Overall weight**	**Overall rank**
Expertise	0.22	Clinical abilities	0.47	Being equipped with clinical knowledge	0.16	4	0.016	
				Ability to make the correct judgment	0.22	3	0.022	
				Possessing clinical handling ability	0.27	1	0.027	10
				Being familiar with clinical ethics, regulations, and systems	0.12	5	0.012	
				Possessing concept of whole medicine	0.23	2	0.023	
		Clinical communication	0.53	Ability to explain the illness to the patient	0.06	7	0.007	
				Ability to use language the patient and his/her family understands	0.14	4	0.016	
				Ability to handle difficult situations	0.09	6	0.010	
				Ability to clearly explain the treatment plan, including risks and benefits	0.13	5	0.015	
				Ability to listen to the patient’s and family’s needs and demands	0.2	1	0.023	
				Ability to be sympathetic to the patient and the family	0.19	2	0.022	
				Ability to communicate and cooperate with the medical team	0.19	2	0.022	
Teaching Ability	0.17	Instructional design	0.27	Ability to set up learning goals	0.20	3	0.009	
				Ability to use proper teaching methods according to learning goals	0.27	2	0.012	
				Ability to arrange proper teaching content according to students’ characteristics	0.53	1	0.024	
		Teaching skills	0.39	Ability to guide students’ thoughts and discussion	0.14	5	0.009	
				Ability to establish a lively and active teaching atmosphere	0.16	3	0.010	
				Ability to provide examples that are close to students’ experience	0.15	4	0.009	
				Ability to give students chances to practice	0.31	1	0.020	
				Ability to introduce clinical cases by empirical medicine method	0.24	2	0.015	
		Teaching material design	0.12	Designing or selecting proper teaching materials by learning goals	0.5	1	0.010	
				Ability to transform clinical cases to teaching materials	0.36	2	0.007	
				Ability to use multimedia teaching materials	0.14	3	0.002	
		Learning evaluation	0.22	Ability to collect related evaluation data	0.14	3	0.005	
				Ability to use proper evaluation tools correctly	0.26	2	0.009	
				Ability to review one’s teaching after evaluation	0.59	1	0.022	
Attitudes and Traits	0.31	Teaching attitudes	0.50	Enthusiastic teaching	0.31	1	0.049	5
				Ability to interact well with students	0.30	2	0.047	6
				Ability to support and help students anytime	0.22	3	0.034	9
				Ability to guide students in their lives	0.18	4	0.028	
		Professional growth	0.24	Ability to absorb new medical information anytime	0.61	1	0.045	7
				Ability to take part in various kinds of teaching workshops	0.22	2	0.016	
				Ability to publish research paper regularly	0.17	3	0.012	
		Personality traits	0.26	Being friendly and kind	0.27	2	0.021	
				Patience	0.46	1	0.037	8
				Being confident in self-expression	0.27	2	0.021	
Beliefs and Values	0.30	Teaching beliefs	0.39	Ability to identify the implications and values of teaching	0.48	2	0.056	4
				Ability to pass on medical knowledge	0.52	1	0.060	2
		Model teaching	0.61	Ability to teach by words and set an example	0.68	1	0.124	1
				Acting as a role model for young teachers	0.32	2	0.058	3

## Discussion

According to Spencer & Spencer, a competency has some components which are visible like knowledge and skills but other behavioral components like attitudes, traits, thinking styles, self-image, organizational fit etc. are hidden or beneath the surface [26]. Our work has successfully built an indicator framework to identify the components of core competency for clinical teachers in Taiwan. Similarly, the results showed that inner characteristics (i.e., attitudes, traits, beliefs, and values) are perceived as more important than surface ones (i.e., professional knowledge, skills, and teaching competency). The finding that 9 out of the top 10 indicators belonged to the two “inner characteristics” dimensions supports the assertion that instruction in areas such as attitudes and affection has a profound influence on students [4,27]. Although such intrinsic abilities had not been confirmed or measured like formal programs, [28] our research stresses the importance of “Attitudes and Traits” and “Beliefs and Values” as core competencies for outstanding clinical teachers.

The perceptions of these teachers in our analysis are remarkably in line with those found in studies in other populations. There have been some frameworks defining outstanding clinical teachers [8-12]. The abilities of clinical teachers can be divided into many aspects, such as teaching skills, [13] interactions with and support to students, [14,15] medical skills (including abilities to effectively demonstrate the medical history taking and physical examination skills, to discuss the recent developments within the field of medicine, and to demonstrate effective interactions with patients at the bedside and decision-making skills in group discussions), [16,17] and even other non-cognitive dimensions. For examples, the doctor-patient relationship, teacher-student relationship, and the development of an environment conducive to learning are all important skills for clinical teachers [17-19]. Clinical teachers not only have to hold a positive attitude toward young doctors and be enthusiastic about their work, [20] but they also have to be self-aware, consciously understand their influence on the resident doctors and students, and establish a good learning atmosphere [19]. Students and young doctors identify enthusiasm, compassion, openness, integrity, and good relationships with patients as attributes they seek in their role models; they are also drawn to senior figures who embody responsibility and status [20].

Sutkin et al. performed a literature review to answer the question, “What makes a good clinical teacher in medicine?” From 4,914 titles over a century, 68 articles were selected for analysis and 480 descriptors were identified. These descriptions, coming from a wide array of methodologies, including essays, surveys, qualitative analyses, and observational studies, but from very few empirical data, were grouped into 49 themes, which were clustered into three main categories: physician, teacher, and human characteristics. The authors thus concluded that excellent clinical teaching, although multi-factorial, transcends ordinary teaching and is characterized by inspiring, supporting, actively involving, and communicating with students [29]. The current research again demonstrated that being an outstanding clinical teacher depended on individual personality traits and teaching style the most, followed by what material was taught. With respect to clinical teaching, four roles and the corresponding abilities should be put into practice: 1) the role of doctor to demonstrate clinical competence; 2) the role of teacher to be enthusiastic about teaching and skilled at questioning and explaining; 3) the role of supervisor to encourage thinking and observe the students; and 4) the role of human to establish good teacher-student and doctor-patient relationships.

When we look into the fine details among sub-dimensions and individual indicators, “model teaching,” especially “the ability to teach by words and set an example” is widely perceived as an important component in medical education. This finding is consistent with previous research results, especially in the teacher-student system of medical education. Teachers’ behaviors and words are what the young doctors and medical students learn and imitate. Not only teachers’ attitudes toward patients, but also their beliefs and values about medical work impact students’ performances in the future [27,30]. Next, “teaching beliefs,” especially “the ability to pass on medical knowledge,” provides teachers with a sense of mission and is important for their identities. In addition, most interviewees mentioned that clinical teachers pass down a collection of experiences and attitudes from generation to generation [28]. Teachers with a sense of mission toward such a teaching belief will generate teaching motivation. Subsequently, the third contributing indicator was “to act as a role model for young teachers.” Most interviewees considered that as clinical teachers, they needed to act in a manner for students to imitate. Duvivier et al. stated that clinical teachers were responsible for being role models for students to imitate [12]. Molodysky (2006) wrote that clinical teachers must have the potential of being role models and suggested that related training courses needed to promote such an ability [28].

Other significantly contributing indicators, including the ability to identify the implications and values of teaching, enthusiastic teaching, the ability to interact well with students, the ability to absorb new medical information anytime, patience, and the ability to support and help students anytime, belong to the dimensions of “Attitudes and Traits” or “Beliefs and Values.” Hsieh thought that the meaning of medical education was to nourish young doctors and resolve patients’ problems [4]. The interviewees of this research also noted that clinical teachers must recognize that teaching influences man’s work, makes young doctors grow, and indirectly helps patients. Only by embracing such a teaching philosophy can teachers remain continuously enthusiastic about teaching. Hsieh, Yang, Yen, and Molodysky all held the same point-of-view that clinical teachers’ enthusiasm and devotion is extremely crucial to core competencies [4,28,31,32]. Most interviewees of this research placed such importance on teaching that they devoted their lives to teaching. Additionally, Beaudoin, Molodysky, and Sutkin all claimed that in addition to having professional medical knowledge and ability, clinical teachers needed to interact well with students [28-30]. In this research, many interviewees shared that as they tried to reach out to students and develop good teacher-student relationships, they received more positive feedback from their students. On the other hand, professional medical abilities must be improved with time to remain in touch with the demands of social change [33]. Many interviewees in this research further shared that as clinical teachers, they absorbed new information by reading medical periodicals and attending medical conferences. Furthermore, patience is also a critical characteristic for clinical teachers; they must constantly teach and remind students to keep an eye on patient safety [34]. Clinical teachers should be equipped with “supervising ability” so as to “support and help students anytime”[28]. Many interviewees in this research added that clinical teachers have to be students’ supporters in their clinical work anytime and promote an environment conducive to learning.

The “clinical handling ability” is the only one among the top 10 indicators belonging to the “Expertise” dimension (generally called clinical or medical skills by most people) [33,35]. Both faculty members (clinical teachers) and residents (students) perceived “having clinical competence” as an important teaching attribute [35]. However, “Teaching Ability” and “Expertise” dimensions were perceived as less important than “Attitudes and Traits” and “Beliefs and Values.” According to the Iceberg Theory, explicit skills can be developed by formal courses, but implicit skills cannot [28,36].

Currently, the content of the existing clinical teachers’ cultivation courses in Taiwan are mostly based on the improvement in expertise and teaching ability. However, intrinsic characteristics such as attitudes, traits, and beliefs were perceived to be more important in shaping a “good” clinical teacher, as the study has shown. Since these intrinsic characteristics are the outcomes of long-term socialization and have to be gradually developed through a long career-developing process, it is difficult to train medical doctors to have these inner abilities in a short period of time. Currently, all doctors in Taiwan will automatically become clinical teachers as long as they work in teaching hospitals. However, not all doctors have the intention to be teachers. Thus, initially, it might be better to recruit medical doctors who have possessed these valued components and their expertise and teaching ability could be obtained through training or formal courses. In the long run, we might need to uncover and enhance “dormant abilities” by incorporating these valued elements into the curriculum early in the medical school so that the awareness of becoming a clinical teacher should be implanted in the early stage of career development in a medical professional.

The strength of the study is that these indicators could not only serve as the self-assessment tool for clinical teachers to enhance or strengthen teaching effectiveness and quality, but also serve as a guideline for career-development facilitators to design teaching workshops or train-the-trainer programs. However, since the participants were obtained from a relatively homogeneous group of Taiwanese excellent teachers in the university medical centers and we only used interview as the major data-collecting method, we suggest that in the future research, the clinical teachers from different levels of teaching hospitals should be included, and other methodologies, such as observation, could also be utilized in order to triangulate the data.

## Conclusion

This study developed the components for and demonstrated the relative importance of the core competencies for clinical teachers in Taiwan. The experts regard intrinsic characteristics (attitudes, traits, beliefs, and values) as more important than surface ones (professional knowledge and skills, and teaching competency). These ideas should foster dialogue for changes among clinical teachers, hospital managers, and the medical community.

## Competing interests

The authors declared no competing interests.

## Authors’ contribution

ATL obtained funding for this project and participated in study concept and design, acquisition of data, analysis and interpretation of data, and drafting of manuscript. JWL involved in data collection, drafting of the manuscript, critical revision and editing of the manuscript, and study supervision. Both authors read and approved the final manuscript.

## Pre-publication history

The pre-publication history for this paper can be accessed here:

http://www.biomedcentral.com/1472-6920/14/75/prepub
